# The floating mass transducer as a microphone—a pilot study

**DOI:** 10.1007/s00106-025-01597-1

**Published:** 2025-06-03

**Authors:** Stefan Kaulitz, Carolina Köstler, Kristen Rak, Rudolf Hagen, Stephan Hackenberg, Mario Cebulla

**Affiliations:** 1 Klinik und Poliklinik für Hals-Nasen-Ohrenheilkunde, Kopf- und Hals-Chirurgie, Universitätsklinikum Würzburg, Würzburg, Germany; 2 HNO Groß und Klein, Praxis Dr. med. Stefan Kaulitz, Friedrich-Stein-Straße 9, 97421 Schweinfurt, Germany

**Keywords:** Middle ear, Prostheses and implants, Middle ear implant, Implantable neurostimulators, Cochlear implant, Mittelohr, Prothesen und Implantate, Mittelohrimplantat, Implantierbare Neurostimulatoren, Cochleaimplantat

## Abstract

**Background:**

This study investigates the inverse use of the Vibrant Soundbridge® Floating Mass Transducer (FMT; MED-EL, Innsbruck, Austria) as a microphone in a pilot test. Should this be applicable, it would open up interesting application possibilities, e.g., as a microphone for a fully implantable cochlear implant.

**Materials and methods:**

Experimental measurements on an ear canal–eardrum model were used to analyze the acoustic properties of the FMT when used as a microphone, including frequency response and sensitivity. The FMT from the Direct Drive Simulation Set was coupled to the artificial eardrum for this purpose.

**Results:**

The results show that the FMT has a usable signal-to-noise performance over the entire frequency range investigated, albeit with a non-linear frequency characteristic. The highest sensitivity was found between 1500 and 2000 Hz.

**Conclusion:**

The study suggests that an FMT optimized for microphone properties could be used as a microphone in the middle ear, which would open up new possibilities for the development of fully implantable hearing systems. Further investigations, in particular measurements on the petrous bone, are required to determine the suitability of the FMT as a middle ear microphone more precisely.

## Abstract (de)

**Hintergrund:**

Die Studie untersucht die inverse Nutzung des Floating Mass Transducer (FMT) der Vibrant Soundbridge® (Fa. MED-EL, Innsbruck, Österreich) als Mikrofon in einem Modellversuch. Sollte dies anwendbar sein, ergäben sich interessante Anwendungsmöglichkeiten z. B. als Mikrofon eines vollimplantierbaren Cochleaimplantats.

**Material und Methoden:**

Durch experimentelle Messungen an einem Trommelfell-Gehörgangs-Modell wurden die akustischen Eigenschaften des FMT bei Verwendung als Mikrofon, einschließlich Frequenzantwort und Empfindlichkeit, analysiert. Der FMT aus dem Direct-Drive-Simulations-Set war hierfür auf dem künstlichen Trommelfell angekoppelt.

**Ergebnisse:**

Die Ergebnisse zeigen, dass der FMT über den gesamten untersuchten Frequenzbereich eine brauchbare Signal-Rausch-Leistung aufweist, allerdings mit einer nichtlinearen Frequenzcharakteristik. Die höchste Empfindlichkeit zeigte sich zwischen 1500 und 2000 Hz.

**Schlussfolgerung:**

Die Studie legt nahe, dass ein auf Mikrofoneigenschaften optimierter FMT als Mikrofon im Mittelohr eingesetzt werden könnte, was neue Möglichkeiten für die Entwicklung vollständig implantierbarer Hörsysteme eröffnen würde. Weitere Untersuchungen, insbesondere Messungen am Felsenbein, sind erforderlich, um die Eignung des FMT als Mittelohrmikrofon genauer zu bestimmen.

Cochlear implants have been the standard in hearing rehabilitation for deafness and profound hearing loss for many years. To date, all systems approved on the market are partially implantable. A fully implantable system would have attractive advantages. An implantable microphone is essential for the development of such systems.

## Middle ear microphones

The floating mass transducer (FMT) is part of the Vibrant Soundbridge® (MED-EL, Innsbruck, Austria) partially implantable active middle ear implant (AMEI). It consists of an approximately 2‑mm-diameter housing containing a magnet and a coil. The FMT is coupled either to the ossicles or directly to the cochlea. Electrical sound signals are converted into mechanical vibrations through excitation with an alternating voltage and transmitted to the inner ear. In Germany, the device is authorized for the hearing rehabilitation of patients with sensorineural, conductive, or combined hearing loss. Many studies have demonstrated the benefits and reliability of this AMEI and thus also of the FMT [1–4].

Various partially implantable cochlear implant systems are available for the treatment of profound hearing loss and deafness. These systems require the use of an external speech processor, which includes a microphone and power supply. A fully implantable system would offer several advantages. For example, the ability to wear these systems continuously—even at night or under special conditions such as when wearing a helmet—would mean continuous auditory support and thus an improved quality of life for users.

Fully implantable hearing systems have already been developed but are not currently being used for various reasons [5–7]. While developments in the field of rechargeable batteries in recent years have resulted in realistic solutions for an implantable, reliable, transcutaneously rechargeable energy source [8], the implantable microphone, which is essential for sound recording, continues to pose a challenge. An implantable microphone must be biocompatible and hermetically sealed. The size and shape must allow for a sensible otosurgical placement and the sound quality should be comparable to that of external microphones [9].

Various positions for implantable microphones have been described. For example, there have already been implant systems developed for placement in the mastoid (Esteem; Envoy, Saint Paul, MN, USA; [10]), subcutaneously (Carina and TIKI; Cochlear, Sydney, Australia; [11]), and in the posterior auditory canal wall (TICA; Implex, Munich, Germany; [7]).

Studies have also investigated the positioning of microphone systems in the middle ear and the cochlea [12–16]. The developments described use a piezoelectric principle or a condenser microphone system to convert sound into an electrical signal. As mentioned earlier, the FMT of the Vibrant Soundbridge® (VSB) is designed to generate and emit sound. In the FMT, a mass moves by induction in an electromagnetic field in the core of a coil. As the principle of induction can also be used in reverse, this structure is similar to that of a moving-coil microphone. This enables the functionality of the FMT to be reversed and to be used for sound recording. The present pilot study investigated this possibility of using the FMT in reverse and its characteristics when used as a microphone.

## Materials and methods

**Fig. 1:**
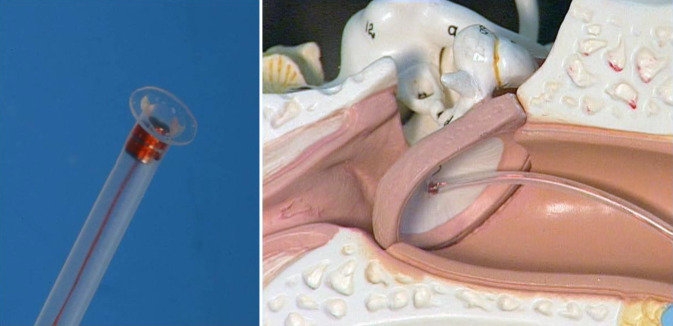
Direct drive simulation test transducer with FMT in the silicone carrier and simulation of the coupling to the eardrum in the model (not to scale)

The so-called direct drive simulation (DDS) refers to a preoperative sound simulation conducted prior to implantation of an AMEI. An FMT encased in a silicone tube (DD-FMT) is coupled to the patient’s eardrum or tympanic membrane (Fig. 1). In this way, realistic sound simulations (speech and music) can be achieved preoperatively [17, 18]. In this study, the DD-FMT used in the DDS as a signal generator was tested in reverse, i.e., to record sounds (microphone). For this purpose, it was connected to an external sound card (Fireface UC, RME Intelligent Audio Solutions, Haimhausen, Germany) via a low-noise preamplifier (LT1115, Linear Technology, Milpitas, CA, USA). The design of the DD-FMT is exactly the same as the FMT used for implantation.

**Fig. 2:**
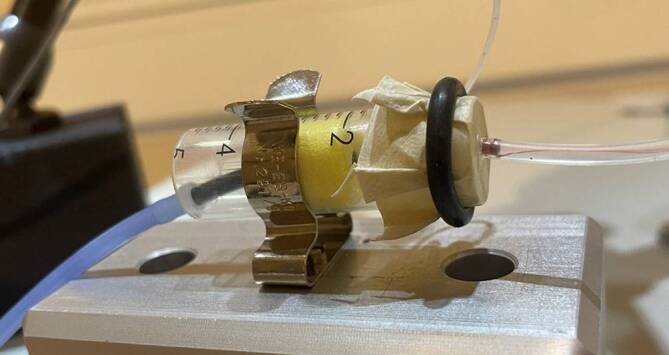
Eardrum auditory canal model with EAR3A, reference microphone, and DD-FMT

To investigate the frequency response of the FMT as a microphone, a simplified model of an ear canal and atympanic membrane was designed (Fig. 2). The ear canal was simulated using a polypropylene/polyethylene syringe (Braun Injekt, Braun, Melsungen, Germany). An ear canal volume of 2 cc was selected, in accordance with the current standards for simulators of the human ear (DIN EN 60318-5:2007-04). An ER3A plug-in earphone (Etymotic, Elk Grove Village, Illinois, USA) was coupled to the foam adapter as a sound generator. A natural drumhead (Roland Meinl Musikinstrumente GmbH & Co. KG, Gutenstetten, Germany), which was stretched using a rubber ring, served as the tympanic membrane. For reference measurement of the sound level in the model, a calibrated microphone (ER-7C, Etymotic, USA) was positioned in front of the artificial eardrum in the ear canal model via a lateral drill hole.

A linear chirp stimulus (100 Hz to 10 kHz) was presented as a test signal for frequency analysis. A sine tone with a frequency of 1 kHz and 94 dB SPL was used to determine the sensitivity of the FMT as a microphone.

The recordings were made in a certified audiometry booth. The DD-FMT was coupled to the membrane of the eardrum ear canal model to record the signal. A drop of castor oil (Caesar & Loretz GmbH, Hilden, Germany) ensured a secure hold on the membrane (Fig. 2). No further fixation between the membrane and the footplate of the DD-FMT was necessary.

The signals from the reference microphone and the FMT were recorded via the external sound card. The incoming analog signal was converted by the sound card’s analog-to-digital converter. The time course of the digital signal was recorded via Matlab (The Math Works, Inc., Natick, MA, USA) as an audio file in wav format with 24 bit and 48 kHz.

## Results

The fit of the insert earphone in the artificial auditory canal and the coupling of the DD-FMT to the eardrum model were stable. No dislocations occurred during the entire measurement procedure (Fig. 2). All hardware and software components of the setup functioned correctly.

**Fig. 3:**
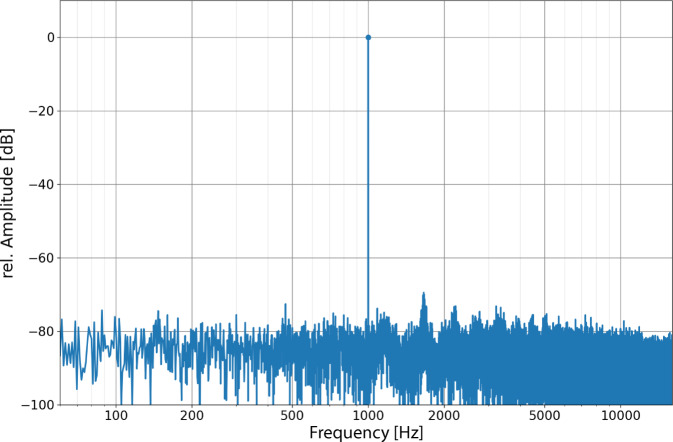
Frequency response of the FMT with a 1-kHz sine tone at 94 dB SPL

The sensitivity of the FMT microphone was determined using a 1-kHz sine tone with a volume of 94 dB SPL. An output voltage of 51 µVrms, corresponding to −26 dB (re 1 mPa), and a signal-to-noise ratio (SNR) of 79.3 dB were determined. The amplitudes were normalized to 0 dB to display the sensitivity and the SNR (Fig. 3).

**Fig. 4:**
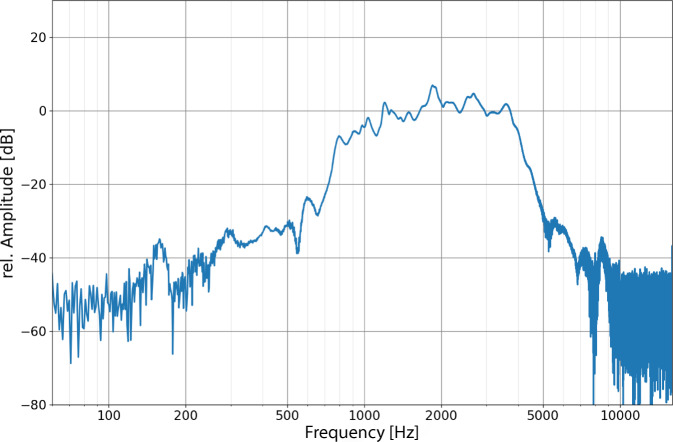
Frequency response of the FMT with a linear chirp (0.1–10 kHz)

In addition, a linear chirp stimulus in the frequency range 0.1–10 kHz was presented via the plug-in headphones to describe the frequency response of the FMT. The frequency response was not linear. The highest sensitivity was found between 1.5 and 2 kHz (Fig. 4). The amplitudes decreased with lower and higher frequencies.

## Discussion

The development of a fully implantable hearing system would represent considerable added value for patients in terms of quality of life. It would allow for almost uninterrupted use (except while charging), even in situations involving exposure to water such as swimming or showering. This would also solve the problem of social stigmatization. Crucial to the development of such systems is an implantable microphone whose technical characteristics, including frequency response and sensitivity, are comparable to microphones currently installed in externally worn speech processors. In our opinion, the middle ear appears to be the optimal location for the placement of such a microphone.

Despite many years of research by various research groups [11–16, 19], it has not yet been possible to develop an implantable middle ear microphone suitable for series production for hearing rehabilitation devices. From the perspective of hearing rehabilitation, a microphone positioned in the middle ear would have particularly interesting acoustic potential. Such a system could capture the natural frequency response and time-of-flight differences in sound resulting from the shape of the pinna and the auditory canal. This would provide patients with additional information that cannot be recorded with external microphones. Disadvantages arise if such a system has faults that can only be corrected by surgery.

Although fully implantable systems have been approved [5–7], they do not use microphones positioned in the middle ear for sound recording. Several publications have described models for middle ear microphones that use either piezoelectric or condenser principles [12, 14, 20]. This study is the first pilot test to investigate the microphone suitability of a mass oscillator that originally worked as an actuator. This mass transducer is already in widespread clinical use an FMT sound transducer of the partially implantable middle ear implant Vibrant Soundbridge®. Since its design is similar to that of a moving coil microphone, the present study investigated its potential use as a sound-recording transducer.

The measurements showed a nonlinear frequency response of the FMT. The greatest sensitivity was between 1500 and 2000 Hz. The highest SNR was found in this range. Above and below this band, the sensitivity decreased significantly. The frequency range above background noise that can still be used for speech processing is between 500 Hz and 6 kHz for the FMT tested.

The sensitivity of the FMT in this study is low at −26 dB rms ref 1 mV. A study of a piezoelectric implantable microphone showed an average sensitivity at 1000 Hz of −44.22 dB rms ref 1 V when coupled to the incus and −53.33 dB rms ref 1 V at the malleus [20].

Gérard et al. [21] investigated the suitability of an implanted microphone for cochlear implants by comparing the hearing results with external standard microphones using pure-tone hearing thresholds. The thresholds in this study were higher for the implanted microphone (44.9 dB) than for the external microphone (36.4 dB). Unfortunately, there is no information on the sensitivity of the microphone systems in dB rms ref 1 V.

### Limitations

There are limitations to the present study. Despite carefully selected materials, it must be assumed that the vibration characteristics of the eardrum–ear canal model do not correspond to the natural vibration behavior of the ear canal and the eardrum. This claim was not made for the first pilot study.

The measured values should therefore always be interpreted with this fact in mind and should not be regarded as real sensitivity and frequency values.

## Practical conclusion

- This study proves that the floating mass transducer (FMT) can in principle be used as a microphone.
- In the eardrum–ear canal model, it had a nonlinear frequency response and low sensitivity.
- In the speech-relevant frequency range of 0.5–6 kHz in this simplified test setup, however, a promising signal-to-noise ratio was present.
- If the low sensitivity and nonlinearity could be compensated for in an implant by appropriate sound processing, it could potentially be used as a middle ear microphone.
- The study encourages further development of a more powerful FMT with a higher sensitivity and subsequent temporal bone model investigations to determine more precisely the frequency response, sensitivity, and background noise.

## Data Availability

The data sets are available from the corresponding author upon reasonable request. The data are kept in a data storage system at the Computing Center of the Department of Otorhinolaryngology, Head and Neck Surgery at Würzburg University Hospital.

## Declarations

For this article no studies with human participants or animals were performed by any of the authors. All studies mentioned were in accordance with the ethical standards indicated in each case.

The supplement containing this article is not sponsored by industry.
